# Inequity in maternal health care service utilization in Gujarat: analyses of district-level health survey data

**DOI:** 10.3402/gha.v6i0.19652

**Published:** 2013-03-06

**Authors:** Deepak Saxena, Ruchi Vangani, Dileep V. Mavalankar, Sarah Thomsen

**Affiliations:** 1 Indian Institute of Public Health–Gandhinagar, Ahmedabad, India; 2 Division of Global Health, Department of Public Health, Karolinska Institutet, Solna, Sweden

**Keywords:** maternal health, health care utilization, inequity, antenatal care, skilled birth attendance, Gujarat

## Abstract

**Background:**

Two decades after the launch of the Safe Motherhood campaign, India still accounts for at least a quarter of maternal death globally. Gujarat is one of the most economically developed states of India, but progress in the social sector has not been commensurate with economic growth. The purpose of this study was to use district-level data to gain a better understanding of equity in access to maternal health care and to draw the attention of the policy planers to monitor equity in maternal care.

**Methods:**

Secondary data analyses were performed among 7,534 ever-married women who delivered since January 2004 in the District Level Household and Facility Survey (DLHS-3) carried out during 2007–2008 in Gujarat, India. Based on the conceptual framework designed by the Commission on the Social Determinants of Health, associations were assessed between three outcomes – Institutional delivery, antenatal care (ANC), and use of modern contraception – and selected intermediary and structural determinants of health using multiple logistic regression.

**Results:**

Inequities in maternal health care utilization persist in Gujarat. Structural determinants like caste group, wealth, and education were all significantly associated with access to the minimum three antenatal care visits, institutional deliveries, and use of any modern method of contraceptive. There is a significant relationship between being poor and access to less utilization of ANC services independent of caste category or residence.

**Discussion and conclusions:**

Poverty is the most important determinant of non-use of maternal health services in Gujarat. In addition, social position (i.e. caste) has a strong independent effect on maternal health service use. More focused and targeted efforts towards these disadvantaged groups needs to be taken at policy level in order to achieve targets and goals laid out as per the MDGs. In particular, the Government of Gujarat should invest more in basic education and infrastructural development to begin to remove the structural causes of non-use of maternal health services.

Millennium development goal (MDG) 5 aims at reducing maternal mortality and improving reproductive health. In India, the maternal mortality ratio (MMR) dropped from 600 deaths per 100,000 live births in 1990 to 390 in 2000 and to 200 in 2010 (1). Despite overall progress, however, wide disparities between different populations exist at the sub-national level, both between and within Indian states. For example, the latest MMR estimates show a gap of 382 deaths per 100,000 live births between Assam (MMR 390) and Kerala (MMR 8) (1).

In India, antenatal care (ANC) services are included in primary health care services for pregnant women and management of the fetus. According to national guidelines, ANC services consist of a set of professional pregnancy checkups, tetanus and other immunizations, prophylaxis through iron and folic acid tablets, blood pressure check-up, advice and information regarding delivery methods and services, nutrition, and postnatal care. The main source of ANC is a network of health centers throughout the country, each serving a population of three to five thousand. These are staffed by trained personnel (auxiliary nurse midwife – ANM) who provide prenatal and postnatal care at the center, who make home visits for pregnant women, help in child delivery and provide immunization services to the infants. Postnatal care (PNC) has a stronger element of hospital-level care compared to antenatal care. PNC services may involve treatment of complications that might have occurred during the delivery and severe health conditions of the newborn, requiring skilled personnel and hospital facilities. The PNC services provided at community level include counseling on family planning, breastfeeding practices, nutrition, management of neo-natal hypothermia, early detection of postpartum complications and referral for such problems. The higher-level health care facilities are intended to provide these services as well as take care of post-delivery complications (2).

A recent systematic review on inequity in maternal health care showed that there are populations in India that seem to be systematically and consistently disadvantaged in Indian society in terms of access to and use of maternal and reproductive health services such as safe delivery and contraceptives (3). These populations – as identified in the published literature – are poor women, the poorly educated, adolescents, and members of scheduled castes (SCs) and scheduled tribes (ST). The findings from this review suggest that poverty causes inequity all over India. However, the review also reflects that residency is less important than economic status. Both the population living in urban slums and the poor living in rural areas have less access to maternal and reproductive health care compared to the non-poor living in the same areas. The findings from this review also suggest that caste and economic status are closely interlinked, with women from marginalized caste groups often also being poor, and thus doubly disadvantaged. It was also documented that the main sources of inequity in maternal health in India – place of residence, education, income, gender norms, and caste – are strong predictors of access to maternal health services (3). For example, in 2009, the rate of institutional delivery in rural areas was 68% compared to 85.6% in urban areas (4). There is also a gap of 36% in skilled delivery rates between rural and urban populations (5). Between 1992 and 2006, use of antenatal care services (ANC) in India increased by 12%, but only 2% of this increase occurred in the poorest wealth quintile (6).

Inequity may also be attributed to differential literacy rates on the basis of place of residence and gender. Currently in India, 65% of the female population is literate compared to 82% among males (7). Female literacy is strongly correlated with maternal health outcomes. In India, 29% of women with no education received at least one antenatal care visit, as opposed to 88% of women with 12 years or more of education (8).

Social class is considered to be the most powerful predictor of health results worldwide. In India, social class is divided along caste and tribal affiliation. The category of ‘scheduled tribe’ is generally the poorest and most disadvantaged in terms of health outcomes, although SCs and ‘other backward castes’ (OBCs) also experience greater levels of social exclusion and marginalization compared to members of the other castes (9). For example, according to the third National Family Health Survey (NFHS-3), access to any ANC during the last birth in the previous five years was only 73.9% for STs, and around 85% for both SCs and OBCs, while it was 95.3% for others (8).

## Gujarat

Strategically located on the West Coast of India, Gujarat is also a gateway to the rich land-locked northern and central parts of the country. Because of its location on the coast, Gujarat also has access to all major port-based countries, including the United Kingdom, Australia, China, Japan, Korea, and the Gulf countries (10).

The population of Gujarat is estimated at 60.3 million, which is approximately 4.99% of the Indian population (11). The literacy rate in Gujarat is on the increase and was 79.31% as per 2011 population census (11). Of that, male literacy stands at 87.23% while female literacy is at 70.73%, a gender gap of 16.5%. Approximately 43% of the population of Gujarat lives in urban areas (11). The overall literacy rate is 79%, although there is a difference between urban (88%) and rural areas (73%) (11).

The data from the 2011 census for the SC and ST populations are still not available in the public domain. According to the 2001 census, SC represented 7.1% of the total population of the state, and ST constituted about 15% of the state population (12). There are 29 notified STs in the state. Gujarat is one of the few states in India having high urban concentration of SC. In 2001, 39.3% of the total SC population was registered in urban areas. While SC is found both in urban and rural areas, the ST population in Gujarat is predominantly rural, with 91.8% residing in rural and 8.2% in urban areas (12).

The MMR in Gujarat is estimated to be 148 per 100,000 live births (1). This is favorable in relation to the India-wide rate of 200. Though Gujarat is an industrially developed state, the MMR of Gujarat is relatively high compared to the states of Tamil Nadu (97/100,000) and Kerala (MMR 8/100,000), whose per capita income is less than Gujarat. In addition, given the disparities in socio-economic measures within the state, and the above-stated importance of these variables in determining maternal health, it is likely that there are significant differences in maternal health outcomes between different population groups within the state. Furthermore, the interaction between structural determinants such as education, caste and income make it difficult for policymakers to identify where the greatest gaps remain in achieving MDG 5, and reducing health inequities, in Gujarat.

Although many studies have used national- and district-level data to analyze the various structural determinants of maternal health, few have attempted to analyze the interaction between these health determinants in order to identify the root cause of health inequity in Indian society (2, 13–15). The purpose of this study was to tease out the relationship between the different sources of inequity in maternal health in the state of Gujarat in order to help policymakers identify the groups that need to be targeted to increase the likelihood of achieving MDG 5 in this state. Analyses are based on the conceptual framework designed by the Commission on the Social Determinants of Health (16).

## Methods

**Fig. 1:**
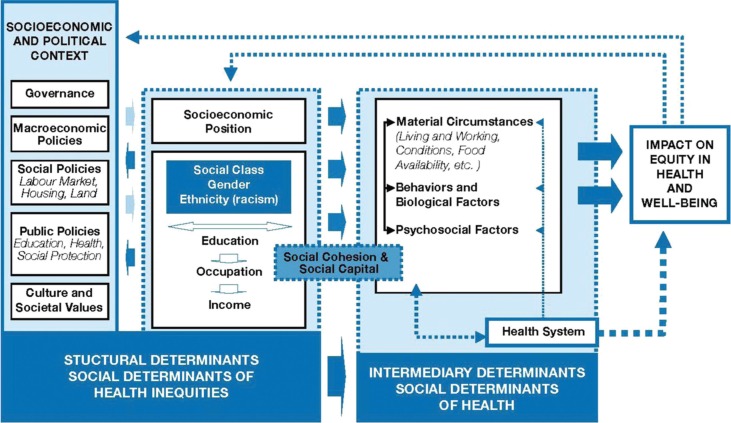
Social determinants of health. Figure based on a model developed by the Commission on Social Determinants of Health (CSDH). Reprinted with permission from WHO.

The analyses in this study are based on a conceptual framework designed by the Commission on the Social Determinants of Health (CSDH), set up by WHO to aid researchers, policy makers, and health planners in their work to reduce health inequity. The determinants include both structural and intermediary determinants (16). The structural determinants include the socio-economic and political context, as well as markers of social position such as education, income, social class (i.e. caste), gender, and ethnicity. The weight and relevance of the assigned social position is influenced by the socio-economic and political context, including governmental policies, cultural values, and the macroeconomic conditions, and its impact on equity in health and well-being is mediated by different intermediary determinants such as living conditions and exposure that directly influence health, as well as access to and quality of care received when encountering the health system. The analyses in this paper are conducted in accordance with this framework, as explained in Figure 1.

### Dataset

We conducted secondary data analysis on raw data of the District Level Household and Facility Surveys (DLHS-3: 2007–2008) for the state of Gujarat. DLHS is a household survey designed to provide information on family planning, maternal and child health, reproductive health of ever-married women and adolescent girls, and utilization of maternal and child health care services at the district level for India. A multi-stage stratified systematic sampling design was adopted for DLHS-3 covering 50 primary sampling units (PSUs) from each district. Information from a total of 24,513 ever-married women age 15–49 years was included in the analyses (17).

### Variables

The structural determinants selected for the present study were education, caste, and wealth index. The intermediary determinants were age, place of residence of mother, and age at marriage of mother. The outcome variables in the present manuscript represent three important components of optimal maternal health care: institutional delivery, use of any modern contraceptive method, and at least three antenatal care visits (the recommended minimum in India). Modern contraceptive methods included sterilization, pills, IUD, injectable, and/or condoms. Institutional delivery included delivery in a hospital, clinic, or at a health center. In the DLHS, wealth index is computed at the national level by combining household amenities, assets, and durables, and then divided into quintiles. In this analysis the poorest and second wealth quintile were grouped into the ‘poor’ category while the non-poor included the top three quintiles. Education status was divided into non-literate, less than 5 years of education and 5 or more years of education.

### Statistical analysis

The analysis was conducted in different stages: first, we conducted descriptive analyses of the women who delivered during the reference period (January 1, 2004–Survey date, 2008). In the second stage, we carried out bivariate regression analysis of predictors for ANC visits, institutional deliveries and use of any modern contraceptive method amongst females in the same sample. Third, using a hierarchical approach recommended by Victora et al. (18), multivariate analysis was conducted to determine odds ratios (ORs) for non-use of services based on selected, significant structural determinants, first without and then controlling for significant intermediate determinants. Finally, in order to examine the relative importance of caste and wealth, we stratified the data and ran the multivariate analyses again using at least three ANC visits as an example, changing the reference group each time. Data were analyzed using SPSS v19.

## Results

### Sample characteristics

Of the 24,513 ever-married women aged 15–49 years who were interviewed for the DLHS 3 in Gujarat in 2007–2008, 7,533 had their last live/still birth since 1 January 2004. Of these, 76.5% lived in rural areas, 37.9% were married before the age of 18, 40.4% were non-literate, 29.1% belonged to STs, 13.2% SC and 36.9% were SEBC (data not shown). The descriptive analysis indicated that those who were 15–24 years old, residing in the urban area, belonging to *non*-ST or SC groups, and having five or more years of education were more likely to have attended at least three ANC visits, to have delivered in an institution, and to be currently using any modern method of contraception at the time of survey were compared to women belonging to other categories (Table 1). This was confirmed in bivariate analyses, which showed that ever married women of 15–24 years, ever-married after 17 years of age, residing in urban areas, belonging to non-poor, other caste, and educated for more than 5 years had a higher utilization of maternal health services (Table 2).

**Table 1:** Utilization of maternal health services and modern contraceptive use among the respondents covered under DLHS-3 who delivered during the reference period

Variable	Category	At least 3 ANC visits	Institutional delivery	Modern contraceptive use	Number of pregnancies*
Intermediary determinants					
Age category	15–24	1,638 (56%)	1,705 (58%)	688 (24%)	2,896
	25–35	2,370 (55%)	2,395 (56%)	1,882 (44%)	4,281
	36–49	126 (35%)	139 (39%)	125 (35%)	356
Age at marriage	<18	1,206 (42%)	1,320 (45%)	974 (34%)	2,852
	>=18	2,928 (63%)	2,919 (63%)	1,722 (37%)	4,682
Place of residence	Rural	2,766 (48%)	2,757 (48%)	1,954 (34%)	5,762
	Urban	1,368 (77%)	1,482 (84%)	742 (42%)	1,772
Structural determinants					
Wealth	Poor	1,204 (62%)	629 (31%)	580 (28%)	2,003
	Non-poor	1,521 (85%)	3,610 (66%)	2,116 (38%)	5,530
Caste group	Scheduled caste	512 (53%)	602 (63%)	360 (38%)	997
	Scheduled tribe	839 (39%)	742 (34%)	635 (29%)	2,193
	SEBC	1,629 (57%)	1,708 (60%)	1,052 (37%)	2,780
	Others	1,104 (74%)	1,129 (75%)	622 (41%)	1,493
Education	Non-literate	1,139 (37%)	1,224 (39%)	958 (31%)	3,043
	Less than 5 years	342 (48%)	343 (48%)	264 (37%)	721
	5 or more years	2,653 (71%)	2,671 (72%)	1,475 (40%)	3,769
Total		4,134 (55%)	4,239 (56%)	4,134 (55%)	7,533

* Unweighted cases.

Total figure may not add to 100 percent due to ‘do not know’ and ‘missing cases’.

Reference period: January 1, 2004 to survey date.

**Table 2:** Bivariate logistic regression analysis of determinants for not receiving/using ANC visits, institutional deliveries and modern contraceptive use amongst females covered under DLHS-3 who delivered during the reference period

		Not receiving ‘at least 3 ANC visits’		Not receiving ‘institutional delivery’		Not using ‘modern contraceptives’	
				
Variable	Category	OR (95% CI)	*p*	OR (95% CI)	*p*	OR (95% CI)	*p*
Intermediary determinants							
Age category	15–24	1 (Referent)	–	1 (Referent)	–	1 (Referent)	–
	25–35	1.03 (0.93–1.13)	0.606	1.1 (1–1.21)	0.049	0.39 (0.35–0.44)	0.000
	36–49	2.32 (1.85– 2.92)	0.000	2.19 (1.75–2.75)	0.000	0.57 (0.45–0.72)	0.000
Age at marriage	<18	2.4 (2.18– 2.63)	0.000	2.03 (1.84–2.23)	0.000	1.17 (1.06–1.29)	0.002
	>=18	1 (Referent)	–	1 (Referent)	–	1 (Referent)	–
Place of residence	Rural	3.68 (3.26– 4.16)	0.000	5.58 (4.87–6.39)	0.000	1.41 (1.26–1.57)	0.000
	Urban	1 (Referent)	–	1 (Referent)	–	1 (Referent)	–
Structural determinants							
Wealth	Poor	4.07 (3.65– 4.54)	0.000	4.32 (3.87–4.81)	0.000	1.58 (1.42–1.77)	0.000
	Non-Poor	1 (Referent)	–	1 (Referent)	–	1 (Referent)	–
Caste group	Scheduled caste	2.43 (2.05– 2.89)	0.000	1.8 (1.51–2.15)	.000	1.18 (1–1.39)	0.052
	Scheduled tribe	4.41 (3.82– 5.09)	0.000	5.82 (5.02–6.74)	0.000	1.71 (1.49–1.96)	0.000
	SEBC	2.1 (1.83– 2.41)	0.000	2.04 (1.78–2.35)	0.000	1.22 (1.07 –1.38)	0.003
	Others	1 (Referent)	–	1 (Referent)	–	1 (Referent)	–
Education	Non-literate	4.29 (3.87– 4.75)	0.000	3.91 (3.53–4.33)	0.000	1.47 (1.33–1.63)	0.000
	Less than 5 years	2.67 (2.27– 3.15)	0.000	2.72 ( 2.31–3.21)	0.000	1.11 (0.94–1.31)	0.204
	5 or more years	1 (Referent)	–	1 (Referent)	–	1 (Referent)	–

### Multivariate analyses

After controlling for structural determinants (wealth, caste, and education status) in the multivariate logistic regression analysis, the odds of *not receiving* ‘at least three ANC visits’ was higher among poor women (OR=2.14; 95% CI=1.89–2.44), STs (OR=2.27; 95% CI=1.93–2.66), SCs (OR=1.92; 95% CI=1.6–2.29), SEBC category (OR=1.5; 95% CI=1.3–1.73), non-literate (OR=3.03; 95% CI=2.71–3.39), or educated less than 5 years (OR=2.25, 1.9–2.67) (Table 3). Similarly, the odds of *not having* ‘institutional deliveries’ was higher among the poor (OR=2.03; 95% CI=1.79–2.31), STs (OR=3.22; 95% CI=2.73–3.78), SCs (OR=1.39; 95% CI=1.16–1.67), SEBC (OR=1.49; 95% CI=1.29–1.72), non-literate (OR=2.74; 95% CI=2.45–3.07), or educated for less than 5 years (OR=2.24; 95% CI=1.89–2.66). *Non-use* of modern contraceptive methods was higher in poor (OR=1.26; 95% CI=1.11–1.44), STs (OR=1.4; 95% CI=1.2–1.63), and non-literate women (OR=1.29; 95% CI=1.15–1.44).

**Table 3:** Multivariate logistic regression analysis of determinants for not receiving/using ANC visits, institutional deliveries and modern contraceptive amongst females covered under DLHS-3 who delivered during the reference period, adjusting for structural determinants only

		Not receiving ‘at least 3 ANC visits’		Not receiving ‘institutional delivery’		Not using ‘modern contraceptives’	
				
Variable	Category	OR (95% CI)	*p*	OR (95% CI)	*p*	OR (95% CI)	*p*
Structural determinants							
Wealth	Poor	2.14 (1.89–2.44)	0.000	2.03 (1.79–2.31)	0.000	1.26 (1.11–1.44)	0.000
	Non-Poor	1 (Referent)	–	1 (Referent)	–	1 (Referent)	–
Caste group	Scheduled caste	1.92 (1.6–2.29)	0.000	1.39 (1.16–1.67)	0.000	1.1 (0.93–1.3)	0.263
	Scheduled tribe	2.27 (1.93–2.66)	0.000	3.22 (2.73–3.78)	0.000	1.4 (1.2–1.63)	0.000
	SEBC	1.5 (1.3–1.73)	0.000	1.49 (1.29–1.72)	0.000	1.12 (0.98–1.27)	0.095
	Others	1 (Referent)	–	1 (Referent)	–	1 (Referent)	–
Education	Non-literate	3.03 (2.71–3.39)	0.000	2.74 (2.45–3.07)	0.000	1.29 (1.15 –1.44)	0.000
	Less than 5 years	2.25 (1.9–2.67)	0.001	2.24 (1.89–2.66)	0.000	1.03 (0.87–1.22)	0.705

All of the variables that were significant in the multivariate analyses controlling for only structural determinants were still significant when controlling for intermediate determinants (age, age at marriage, and place of residence), although the ORs were slightly diminished (Table 4).

**Table 4:** Multivariate logistic regression analysis of determinants for not receiving ANC visits, institutional deliveries and modern contraceptive amongst females covered under DLHS-3 who delivered during the reference period, after controlling for both structural and intermediary determinants

		Not receiving ‘at least 3 ANC visits’		Not receiving ‘institutional delivery’		Not using ‘modern contraceptives’	
				
Variable	Category	OR (95% CI)	*p*	OR (95% CI)	*p*	OR (95% CI)	*p*
Intermediary determinants							
Age category 15–25	1 (Referent)	–	1 (Referent)	–	1 (Referent)	–	
	26–35	1.14 (1.02–1.26)	0.000	1.25 (1.12–1.39)	0.000	0.38 ( 0.34–0.42)	0.000
	36–49	1.66 (1.29–2.14)	0.019	1.56 (1.21–2.01)	0.001	0.46 (0.36–0.59)	0.000
Age at	<18	1.56 (1.4–1.74)	0.000	1.28 (1.15–1.43)	0.000	0.86 (0.77–0.95)	0.004
Marriage	>=18	1 (Referent)	–	1 (Referent)	–	1 (Referent)	–
Place of	Rural	2 (1.75–2.3)	0.000	3.04 (2.62–3.52)	0.000	1.1 (0.97–1.24)	0.125
Residence	Urban	1 (Referent)	–	1 (Referent)	–	1 (Referent)	–
Structural determinants							
Wealth	Poor	1.87 (1.64–2.13)	0.000	1.72 (1.51–1.95)	0.000	1.31 (1.15–1.5)	0.000
	Non-Poor	1 (Referent)	–	1 (Referent)	–	1 (Referent)	–
Caste group	Scheduled caste	1.75 (1.46–2.11)	0.000	1.25 (1.03–1.51)	0.022	1.04 (0.87–1.23)	0.681
	Scheduled tribe	1.87 (1.58–2.21)	0.000	2.55 (2.16–3.02)	0.000	1.24 (1.06–1.46)	0.008
	SEBC	1.35 (1.17–1.57)	0.000	1.33 (1.14–1.55)	0.000	1.03 (0.9–1.18)	0.627
	Others	1 (Referent)	–	1 (Referent)	–	1 (Referent)	–
Education	Non-literate	2.5 (2.23–2.81)	0.000	2.29 (2.03–2.57)	0.000	1.41 (1.25–1.59)	0.000
	Less than 5 years	1.94 (1.63–2.31)	0.000	1.93 (1.62–2.3)	0.000	1.08 (0.91–1.29)	0.385
	5 or more years	1 (Referent)	–	1 (Referent)	–	1 (Referent)	–

### Stratified multivariate analysis

In the stratified analyses, being poor was associated with less utilization of ANC services independently of caste category (Table 5). Women who were poor among the ‘other’ caste category were 5.65 times (95% CI=3.36–9.48) less likely to use ANC services than the non-poor in the same caste category. Poor women belonging to an ST were 5.32 times (95% CI=4.43–6.4) less likely to use ANC services than the non-poor advantaged groups. Similarly women in the poor SC category were 5.1 times (95% CI=3.54–7.36) less likely to utilize ANC services than the non-poor ‘others’. There were no differences between the poor in the different caste groups. However, there was evidence of effects on ANC use due to caste status among the non-poor. Women belonging to an ST, but not poor were 2.37 times (95% CI=1.97–2.85) less likely to use ANC services than the non-poor advantaged groups (‘other caste’).

**Table 5:** Multivariate logistic regression analysis of ‘at least 3 ANC visits’ (not receiving) and wealth within different caste groups (adjusted for education), amongst females covered under DLHS-3 who delivered during the reference period

Caste	Wealth	Yes	No	OR (95% CI)	p	OR (95% CI)	p	OR (95% CI)	p	OR (95% CI)	p	OR (95% CI)	p
ST	Poor	342 (27%)	916 (73%)	5.32 (4.43–6.4)	0.000	0.94 (0.56–1.58)	.822	3.14 (2.69–3.67)	0.000	2.67 (2.19–3.25)	0.000	2.25 (1.86–2.72)	0.000
	Non-poor	497 (55%)	408 (45%)	2.37 (1.97–2.85)	0.000	0.42 (0.25–0.7)	.001	1.4 (1.19–1.64)	0.000	1.19 (0.97–1.45)	0.091	1 (Referent)	
SC	Poor	48 (28%)	123 (72%)	5.1 (3.54–7.36)	0.000	0.9 (0.49–1.65)	.743	3.01 (2.12–4.29)	0.000	2.56 (1.76–3.72)	0.000	2.16 (1.49–3.12)	0.000
	Non-poor	464 (59%)	323 (41%)	1.99 (1.64–2.42)	0.000	0.35 (0.21–0.6)	.000	1.18 (0.99–1.4)	0.060	1 (Referent)		0.84 (0.69–1.03)	0.091
SEBC	Poor	210 (40%)	318 (60%)	2.76 (2.21–3.46)	0.000	0.49 (0.29–0.83)	.008	1.63 (1.34–2)	0.000	1.39 (1.1–1.75)	0.006	1.17 (0.93–1.47)	0.184
	Non-poor	1,420 (61%)	908 (39%)	1.69 (1.45–1.97)	0.000	0.3 (0.18–0.5)	.000	1 (Referent)		0.85 (0.72–1.01)	0.060	0.72 (0.61–0.84)	0.000
Other	Poor	21 (24%)	66 (76%)	5.65 (3.36–9.48)	0.000	1 (Referent)		3.34 (2.01–5.54)	0.000	2.83 (1.68–4.77)	0.000	2.39 (1.42–4.01)	0.001
	Non-poor	1,083 (77%)	330 (23%)	1 (Referent)		0.18 (0.11–0.3)	.000	0.59 (0.51–0.69)	0.000	0.5 (0.41–0.61)	0.000	0.42 (0.35–0.51)	0.000

Being poor was also associated with less utilization of ANC services irrespective of the place of residence (Table 6). Rural poor and urban poor were 5.22 (4.45–6.13) and 5.19 (3.02–8.94) times less likely to utilize the ANC services respectively in comparison to the urban non-poor.

**Table 6:** Multivariate logistic regression analysis of ‘at least 3 ANC visits’ (not receiving) and wealth and place of residence (adjusted for education), amongst females covered under DLHS-3 who delivered during the reference period

Place of residence	Wealth	Yes	No	OR (95% CI)	*p*	OR (95% CI)	*p*
Rural	Poor	599 (30%)	1,388 (70%)	5.22 (4.45–6.13)	0.000	2.21 (1.96–2.5)	0.000
	Non-poor	2,167 (57%)	1,624 (43%)	2.36 (2.06–2.71)	0.000	1 (Referent)	–
Urban	Poor	22 (34%)	42 (66%)	5.19 (3.02–8.94)	0.000	2.2 (1.29–3.75)	0.004
	Non-poor	1,346 (79%)	363 (21%)	1 (Referent)	–	0.42 (0.37–0.49)	0.000

## Discussion

The data from district-level health surveys in the relatively wealthy state of Gujarat indicate that the most significant source of inequity in relation to use of maternal health services in the population is poverty. The poor, regardless of caste, are the most disadvantaged group in relation to maternal health services in Gujarat. Furthermore, our hierarchical analyses show that the effects of poverty and caste on use of maternal health services are independent of age of mother, age at marriage (before or after 18), and place of residence. This indicates that it is not sufficient to explain the differences because the poor are more likely to marry young or to live in rural areas.

In addition to poverty, social class – measured as caste in India – is also an important structural determinant of inequity in maternal health in Gujarat. Our equity analyses show that if you are non-poor, and from a caste other than general (‘others’), you are also disadvantaged in relation to access to maternal health services. This reiterates the central importance that social class plays in determining inequities in health (16). There are several theories about why class is an important structural determinant of health and health inequity, such as the role of social class in influencing political decision-making, which in turn has an effect on policies that affect education, health care, and employment. The implications are that it is not enough to focus on adjusting the health system. Even introducing and strengthening ‘demand-side’ interventions designed to encourage use of maternal health services, such as conditional cash transfers and voucher schemes will not be enough to affect changes in use of services in some groups (19). More comprehensive structural changes are needed to address other factors such as gender and social norms, political influence, corruption, and discrimination (3).

## Policy implications

The results of this paper indicate that it is not unrealistic to assume near universal use of maternal health services *if the structural determinants identified above are addressed*. Women who are urban, literate, wealthier, younger, ever-married at 18 or above use services more than their rural, illiterate, older, and ever-married younger counterparts. Over 80% of urban women are delivering in institutions. Similarly, over 80% of the non-poor attended at least three ANC visits (the recommended minimum in India) during their last pregnancy. Educational levels also show an apparent ‘dose-response’ relationship with use of all maternal health services studied here. That is, the more education one has, the more likely one is to use ANC, institutional delivery, and modern methods of contraception. The relationship between mother's education and use of maternal health services is already well documented (8). What is significant for policymakers to note here is that the high levels of use among the wealthy, urban, and educated women indicate *what is possible to achieve in Gujarat*. Furthermore, our study results show that there are fewer disparities in access to family planning services amongst the disadvantaged populations. This also begs the question ‘what are the family planning programs doing right that other maternal health programs are not?’ It also indicates that it should be possible to reach the poor and disadvantaged classes with other maternal health services.

As mentioned in the introduction, Gujarat has experienced rapid growth and is one of the wealthiest states in India and also showing improvement in overall health status. However, improvements in the health of the general population do not lead to the removal of disadvantage in society (20, 21). To achieve the desired targets under MDGs in an equitable manner, there is an urgent need to review the existing policies implemented by the state to reduce such health inequalities. Furthermore, the State of Gujarat should also design systems to monitor equity (22).

Greater attention needs to be directed towards the assessment of health deprivation among the poor. Availability alone may not be sufficient, unless it is supported by a policy of greater subsidization of health facilities through special schemes for maternal health care. Targeted interventions need to be initiated for better delivery of services and tools directly to those who are in greatest need. Additionally, decisions on resource allocation for public health need to be taken along with other pertinent factors, which will affect the efficacy of the policy matrix in total, such as poverty rates and education. Perhaps the state should recognize the fact that expenditures on health and education are complementary in nature and, if combined, will produce large individual and social benefits. In this sense, this analysis may help the policy planners and hopefully provide a road map for the path ahead.

## Conclusions

Poverty is the most important determinant of non-use of maternal health services in Gujarat, with poor women bearing the brunt of health disadvantage, above and beyond social caste or place of residence. Social class is also an important determinant of health inequity in access to maternal health for the non-poor. More concerning is the fact that India is experiencing an increasing rich poor gap which may further increase challenges to the overall progress towards the achievement of the MDGs. This analysis also reveals that there is a strong association between the structural determinants identified and that these interact when influencing access to optimal maternal care.

The high levels of use of maternal health services among some, but not all, populations of Gujarat indicate that structural determinants of health – income, education, social position, and place of residence – are the driving forces behind the inability to reach targets in MDG 5 in this relatively wealthy state. Therefore, the Government of Gujarat is encouraged to invest in education and infrastructural development on a much wider scale. Greater knowledge of the barriers to accessibility, availability, acceptability, and quality of maternal health services for the poor, rural, and tribal populations is also needed.
